# Non-Cicatricial Alopecia and Its Association with Anthropometric Measurements and Nutritional Laboratory Markers

**DOI:** 10.3390/life14050609

**Published:** 2024-05-09

**Authors:** Katarzyna Wróblewska-Kończalik, Mariola Pawlaczyk, Jerzy Kolasiński, Małgorzata Kolenda, Izabela Miechowicz, Agnieszka Seraszek-Jaros, Anna Kroma-Szal, Justyna Gornowicz-Porowska

**Affiliations:** 1 Kolasiński Clinic, Hair Clinic Poznan, 62-020 Swarzędz, Poland; kasia19711@wp.pl (K.W.-K.); magy1970@me.com (M.K.); 2 Department and Division of Practical Cosmetology and Skin Diseases Prophylaxis, Poznan University of Medicinal Sciences, Rokietnicka 3, 60-806 Poznań, Poland; mariolapawlaczyk@ump.edu.pl (M.P.); justyna.gornowicz-porowska@ump.edu.pl (J.G.-P.); 3 Department of Computer Science and Statistics, Poznan University of Medical Sciences, 60-806 Poznań, Poland; iza@ump.edu.pl; 4 Department of Bioinformatics and Computational Biology, Poznan University of Medical Sciences, Bukowska 70 Street, 60-812 Poznań, Poland; seraszek@ump.edu.pl

**Keywords:** hair loss, diet, biochemical parameters, anthropometric parameters

## Abstract

Alopecia constitutes one of the most common dermatological disorders, and its steadily increasing prevalence is a cause for concern. Alopecia can be divided into two main categories, cicatricial/scarring and non-cicatricial/non-scarring, depending on the causes of hair loss and its patterns. The aim of this study was to investigate the relationship between anthropometric and nutritional laboratory parameters in Caucasian adult women and men with non-cicatricial alopecia. A total of 50 patients (37 with non-cicatricial alopecia and 13 healthy controls) were included in the study. Clinical examination and scalp trichoscopy were performed. The anthropometric and nutritional laboratory parameters were collected and analyzed. No statistically significant differences in the laboratory findings were found. The patients with non-cicatricial alopecia were statistically significantly younger as compared to the controls. An elevated risk of hair loss, which was detected among the younger participants, might be associated with a modern lifestyle and the so-called ‘Western diet’. It seems safe to assume that suboptimal nutrition and poor eating habits during childhood might constitute risk factors for early hair loss.

## 1. Introduction

Alopecia is a catch-all term which encompasses various types of hair loss, affecting the scalp or even the entire body. The condition may be transient or permanent [1] and has been reported to affect individuals of all ages and genders. Alopecia remains one of the most common dermatological disorders, and its increasing global prevalence is an ongoing cause for concern [2].

The growth of hair follicles is cyclical. Hair growth is a continuous process which consists of the following phases: anagen—growth; catagen—regression; telogen—rest; and exogen—shedding [3]. Individual hair follicles cycle independently, with each hair follicle undergoing ten to thirty cycles in the course of one lifetime [3]. Anagen is a highly mitotic, active phase which is characterized by the production of a hair shaft from the hair follicle, whereas catagen and telogen are characterized by a transition into quiescence, describing the regression and the resting phase of the follicle, respectively, ultimately resulting in hair shedding. Therefore, if anagen shifts to the resting phase prematurely, excessive shedding and thinning can occur, which might lead to alopecia. Conversely, a reduction in the number of hair follicles residing in the telogen phase manages hair loss. Lastly, exogen describes the termination of telogen and the initiation of anagen. Nutritional imbalance is a factor which increases anagen-to-telogen transition and may be predictive of hair loss [3].

Depending on the hair loss causes and patterns, alopecia can be classified into two main categories: cicatricial/scarring and non-cicatricial/non-scarring [4]. In cicatricial alopecia, which is the less prevalent type of the disorder, the hair follicles are irreversibly damaged, leading to permanent hair loss [4]. In non-cicatricial alopecia, the capillary cycle is altered but the hair follicles are preserved, with potential for hair regrowth [4]. 

The types of non-cicatricial alopecia include androgenetic alopecia (AGA), also known as male pattern hair loss (MPHL) and female pattern hair loss (FPHL); telogen effluvium (TE); and alopecia areata (AA). Androgenetic alopecia typically develops when dihydrotestosterone (DHT), a potent testosterone metabolite, binds to the androgen receptors expressed in the hair bulb and papilla, located in the hair follicle, which results in miniaturization of the hair follicles [5]. Telogen effluvium is characterized by excessive hair shedding, often with accompanying trichodynia and hair dryness or a lighter hair tone [6]. In alopecia areata, sudden focal hair loss appears as a result of a triggering agent [7].

The etiology of alopecia includes genetic factors, hormonal imbalance, infection, environmental triggers, exposure to chemicals, medicines, nutritional deficiency, extreme stress, and idiopathic causes [8,9]. It is crucial to identify the cause of hair loss in order for the treatment to be effective.

The data [4] presented in Table 1 detail the types and etiologic factors of non-cicatricial alopecia.

Although the data about the links between lifestyle habits and hair loss remain inconsistent, recently, the role of nutrition in hair loss has been extensively discussed in the literature. Imbalanced nutrition and poor eating habits have been identified as causative factors for alopecia [3,10,11,12].

Proper nutrition is vital in order for anagen and telogen to have balance, and caloric or nutritional deficiency can negatively impact not only hair growth but also hair structure and pigmentation [3,10]. The literature offers conflicting reports about the merits of nutritional supplementation for hair loss, especially among non-deficient individuals, and this topic has generated much debate in recent years. Moreover, the over-supplementation of some nutrients may increase toxicity and even contribute to hair loss [3]. Cell division, which is a process that requires the energy supplied by food, takes place within the hair follicle. The quality of the absorbed nutrients affects the hair structure and the tempo of its growth. Micro- and macro-element deficiency, resulting from a poorly balanced diet, malabsorption, eating disorders, or poor eating habits, may lead to hair loss [11]. Excessive consumption of simple carbohydrates and processed foods also contributes to hair loss [12]. Regardless, evidence to support the correlation between non-cicatricial alopecia and metabolism-related indicators remains inconclusive.

The aim of this study was to investigate possible relationships between anthropometric and nutritional laboratory parameters in Caucasian adult women and men with non-cicatricial alopecia. 

## 2. Materials and Methods

### 2.1. Study Population and Diagnostic Criteria

**Figure 1:**
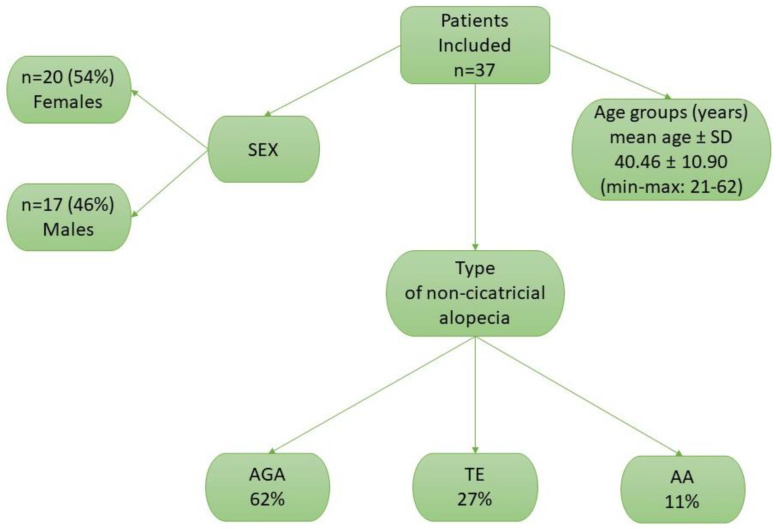
Patient distribution by sex, age, and type of non-cicatricial alopecia in the study group. Abbreviations: n—number of patients; AGA—androgenetic alopecia; TE—telogen effluvium; AA—alopecia areata; SD—standard deviation.

A total of 50 participants (Caucasian, of Polish nationality) were included in the study. The patients were subdivided into two groups: 37 with non-cicatricial alopecia (AGA, TE, AA) —the study group (mean age 40.46 ± 10.90 years)—and 13 healthy controls (mean age 50.69 ± 8.41 years)—the control group. The patient characteristics are presented in Figure 1 and Table 2.

All the patients were evaluated at the Kolasinski Hair Clinic. The diagnoses of AGA, TE, and AA were based on clinical examination and scalp trichoscopy. The inclusion criteria for the study group were as follows: (1) AGA was clinically suspected in cases of frontal, diffuse central, or vertex/frontal accentuation with sparing of the occiput; (2) clinical diagnosis of TE was based on detailed examination of the scalp, which revealed an increased percentage of short anagen hairs growing close to the scalp; (3) AA was diagnosed if empty hair follicles, black dots, and dystrophic hairs, broken hairs, and exclamation point hairs were found on trichoscopy [13].

**Figure 2:**
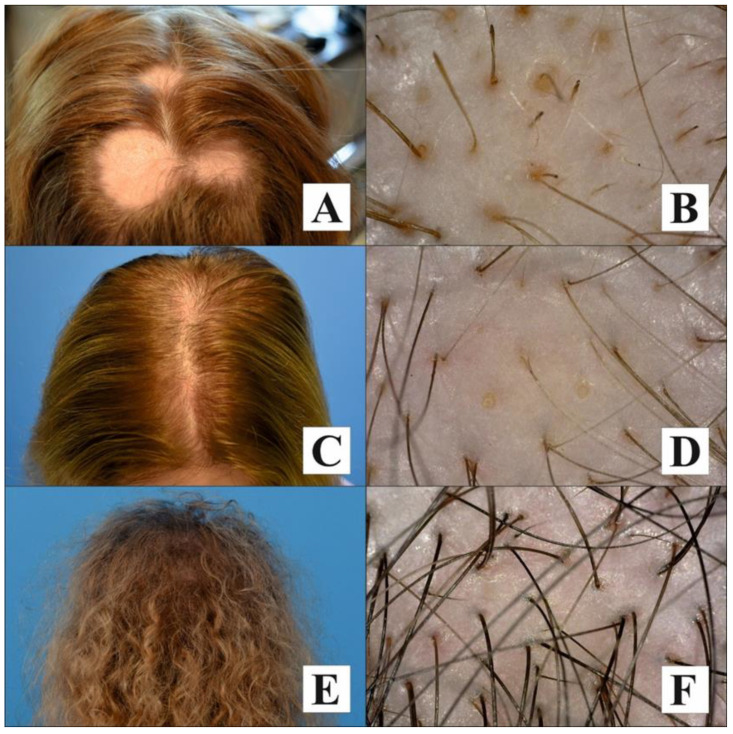
Clinical and trichoscopic (Dino-Lite TrichoScope Polarizer × 50) presentation of various clinical types of non-cicatricial alopecia: (**A**) a middle-aged woman with alopecia areata presenting exclamation point hairs and yellow dots (**B**); (**C**) a middle-aged woman with androgenetic alopecia presenting hair follicle miniaturization and empty follicles (**D**); (**E**) a middle-aged woman with telogen effluvium presenting predominance of single follicular units and vertically growing hair (**F**).

Images from the study group patients are presented in Figure 2.

The negative control group included healthy individuals with no history of hair plucking. The inclusion criteria for the negative control group were as follows: (1) no family history of alopecia, (2) negative result of the pull test.

Trichoscopic imaging of the scalp was performed using a FotoFinder^®^ Dermoscope II camera (Teach Screen software GmbH; Bad Birnbach, Germany) (at 20- and 70-fold magnification) in all the patients. Also, all the individuals underwent a gentle pull test. The Hamilton–Norwood scale and the Ludwig scale were used for male and female patterns of hair loss, respectively. Trichoscopy was performed in the control group to confirm the absence of alopecia symptoms.

### 2.2. Measurements

Peripheral blood samples were obtained from each individual upon admission (baseline). The samples were centrifuged for 10 min at 3500 rpm and stored at −20 °C until needed. The biochemical analyses included the following parameters: hemoglobin (Hb), white blood cells (WBC), average volume of erythrocytes (MCV), total cholesterol (TC), triglycerides (TG), ferritin, transaminases (AST, ALT), glucose, vitamin B_12_, urea, creatine, acute-phase protein (CRP), thyroid-stimulating hormone (TSH), and dehydroepiandrosterone sulfate (DHEAS). ELISA was used to measure the serum level of anti-tissue transglutaminase (tTG) IgA. A one-step delayed-action immunoassay, a fully automated technology in the Alinity I system (Abbott Laboratories, Abbott Park, IL, USA), was used for the quantitative determination of 25(OH)D. The body mass index (BMI) and the anthropometric measurements (weight, height, and waist circumference) were calculated.

### 2.3. Statistical Analysis

Statistical analysis was conducted using Statistica 13.3 (TIBCO Software Inc, Palo Alto, CA, USA) and PQStat v.1.8.6.120 (PQStat Software, Poznan, Poland). The Shapiro–Wilk test was used to check the normality of the variable distribution. To compare the results of the laboratory parameters between the study group and the controls, Student’s *t*-test was used if the data were distributed according to a Gaussian curve. The Mann–Whitney U test was used for variables without a normal distribution. A *p*-value of <0.05 was considered statistically significant.

The study was approved by the local ethics committee (Poznan University of Medical Sciences, Poland; no 112/19).

## 3. Results

Demographic and anthropometric data, as well as detailed patient characteristics, are presented in Table 3. A statistically significant difference in patient age was observed. The patients with non-cicatricial alopecia were statistically significantly younger than the controls.

No statistically significant differences were found between the study group and controls as far as the mean values of the laboratory parameters were concerned. In the study group, we found no cases of anemia, and the hemoglobin concentration was normal, but low levels of ferritin were detected in as many as 18 (48.65%) patients. Among the patients with alopecia, abnormal TSH levels were found in 3 patients (8.1%): elevated in 2 and decreased in 1 case. Increased TC levels were observed in 20 (54.05%) and elevated TG in 10 (27.2%) patients. Decreased vitamin D levels were found in 64.86% of the study group.

The results of the laboratory tests are presented in Table 4.

## 4. Discussion

In this study, we investigated possible relationships between nutritional status, assessed using anthropometric measurements and biochemical parameters, and non-cicatricial alopecia. While interpreting these findings, one should keep in mind the limitations of our study. We present just the prospective experience of a single referral center with recruitment during the COVID-19 pandemic, hence the relatively small number of patients. Still, this limitation did not prevent the appropriate statistical analysis, enabling the interpretation of our data.

The role of biochemical parameters in the development of alopecia has been reported [12,14,15,16]. The prevalence of non-cicatricial alopecia continues to increase due to unfavorable changes in patient lifestyle, nutrition, and cosmetic habits, as well as hormonal disbalance or psychological stress [14]. Hair loss is typically reported in older populations, which is associated with the hormonal hypothesis of the stimulating role of estrogen in the growth hair cycle. In our study, alopecia was observed in younger patients as compared to healthy controls.

Shi et al. [12] reported that the consumption of sugar-sweetened beverages was associated with an elevated risk of hair loss in young men. Alopecia has been known to be linked with glucose metabolism, and high sugar intake may play a role in hair loss by activating the polyol pathways [15,16]. In our study, no abnormal levels of glucose were detected in the study group or the controls. The biochemical symptoms of some types of non-cicatricial alopecia (e.g., AGA) in the scalp are highly suggestive of an overactive polyol pathway [12]. Also, personal distress may play a crucial role in hair loss in young people [16]. The patients with non-cicatricial alopecia in our study were statistically significantly younger than the controls.

Some authors have demonstrated an association between non-cicatricial alopecia, especially AGA, and a high risk of metabolic syndrome (MS), as those patients presented with significantly poorer metabolic profiles [17,18,19,20]. In our study, a positive correlation was not observed between AGA and MS, probably because the investigated AGA patients were relatively young. In a population of Chinese females with AGA, Zhu et al. discovered that a greater waist circumference, hip circumference, and waist–hip ratio were associated with an increased risk of hair loss as compared to controls [17]. Qui et al. found that patients with AGA had significantly poorer metabolic profiles in terms of BMI, waist circumference, fasting glucose, blood lipids, and blood pressure [18].

In our study, similar mean WHtR values were observed in both groups (0.49 and 0.51 in the study group and controls, respectively). Still, a higher WHtR ratio (>0.5) was detected in the control group, which is consistent with the findings of Danesh-Shakiba et al. [21], who analyzed anthropometric parameters, hypertension, and smoking in AGA individuals and controls. In our study, a higher risk of cardiovascular disorders and metabolic diseases was detected among the controls (WHtR > 0.5—controls). Concentrations of plasma urea and creatinine, the end products of protein metabolism, are indices of proper nutrition or risk of malnutrition or renal diseases. In our study, no abnormal levels of these parameters were detected in the study group or the controls.

Various authors have focused their attention on vitamin and mineral deficiencies in patients presenting with hair loss [22,23,24,25,26,27,28,29]. The relationship between low serum vitamin D levels and the development of alopecia areata and telogen effluvium is the reason why vitamin D supplementation should be recommended to affected individuals [22,29]. Also, successful treatment of alopecia areata with topical calcipotriol has been reported by Kim and Lee [23]. According to some authors, vitamin D alters cytokine production and affects epithelial cell differentiation [22,23], and its deficiency is positively correlated with hair loss [11,24] in patients with alopecia areata. Vitamin D activates the Vitamin D Receptor (VDR), which is important for the expression of genes responsible for anagen initiation [25,26]. In humans, mutations in the VDR gene lead to impaired vitamin D synthesis, resulting in systemic and bone disorders, as well as hair thinning and hair loss [20], which is consistent with our findings. In our study, vitamin D insufficiency was confirmed in as many as 64.86% of the patients from the study group. 

A variety of hormones, e.g., thyroid hormones, dehydroepiandrosterone (DHEA), DHT, estrogens, and testosterone, have been shown to affect the hair cycle and hair growth [3]. Elevated DHEAS levels were observed in men aged <35 years with early-onset AGA, defined as alopecia over grade III (according to the Hamilton–Norwood scale), as compared to controls [30]. Therefore, early-onset AGA has been investigated as a clinical sign of the male equivalent of polycystic ovarian syndrome (PCOS) [30]. In our study, we found higher levels of DHEAS in patients with non-cicatricial alopecia as compared to the control group (263.66 ± 134.95 vs. 193.42 ± 78.44, respectively). However, the difference was not statistically significant. Hair loss continues to be one of the well-known symptoms of thyroid dysregulation, in cases of both deficient and excessive levels of thyroid hormones [31,32,33]. It is postulated that thyroid hormone signaling is necessary for correct stem cell activation from the hair bulge, and improper stem cell signaling may be associated with hair loss. Moreover, prolonged thyroid hormone stimulation is probably linked with progenitor cell differentiation and subsequent stem cell depletion. Thus, it seems that thyroid-stimulating hormones and thyroxine levels should be measured as part of the standard work-up in patients with non-cicatricial alopecia.

Interestingly, some authors have investigated and reported an association between ferritin deficiency and hair loss [34,35]. Öner et al. observed low serum ferritin, the storage form of iron, in patients with non-cicatricial alopecia, especially among women. In our study, however, we did not find such an association. Although the exact mechanism of how a low ferritin concentration leads to hair loss has not been fully elucidated, one theory emphasizes the role of iron as a cofactor for ribonucleotide reductase. This iron-dependent enzyme is also the rate-limiting enzyme for the biosynthesis of deoxyribonucleotides (dNTPs), which are essential for DNA replication and DNA damage repair [36]. Since hair follicle cells divide rapidly, they require constant use of ribonucleotide reductase. Iron deficiency may limit the efficiency of this enzyme, which, in turn, might lead to decreased cell turnover and regeneration, impeding hair growth [3]. Therefore, it is advised to measure serum iron levels in non-cicatricial alopecia patients. Nevertheless, the findings of our study did not confirm this observation. No cases of anemia were observed in the study group. However, low ferritin levels were detected in 18 individuals, accounting for 48.65% of the study group population. Rasheed et al. [27], in their study on the relationship between ferritin and vitamin D concentrations and TE, as well as FPHL, confirmed the role of ferritin in AA. The VDR regulates the expression of genes affecting stem cell proliferation in the hair follicle, and proper iron and ferritin concentrations regulate transcription factors in the epithelial tissue [27]. Gowda et al., in their cross-sectional study, confirmed a link between low ferritin and iron concentrations and hair loss, especially among patients with TE (20.37%), as compared to individuals with AGA (AA—2.94%, FPHL—16.67%) [37]. Their findings were not confirmed by other authors [24,38,39].

The role of vitamin B_12_ in the hair growth cycle has been suggested by various authors [28,29,34]. Tamer et al. did not observe any differences in the serum concentration of vitamin B_12_ between their group of patients with diffuse hair loss (study group) and healthy controls, which is consistent with our observations. In our study, no statistically significant differences in the mean serum vitamin B_12_ concentrations were found between the patients with non-cicatricial alopecia and the healthy controls (405.62 ± 195.72 vs. 429.31 ± 128.62, respectively). Similar findings were reported also by Cheung et al. [29]. In a retrospective cross-sectional study, conducted in 413 patients diagnosed with telogen effluvium, these authors reported that only 2.6% of the patients presented with vitamin B_12_ deficiency.

In our study group, anti-tGT IgA antibodies were found only in the patients with AA, but these levels did not exceed the normal ranges, so they do not seem to be an etiological factor.

Some studies have reported a correlation of hypertension and hyperlipidemia with the risk of hair loss, especially androgenous alopecia [38], which was not confirmed in our study. An elevated TG concentration was found in 27.2% of our study group. The mean TG concentration in the control group was 110.84 mg/dL (±34.35). Total cholesterol was elevated in both groups. Diet and poor eating habits may be causes of lipid dysregulation [29,40]. Furthermore, genetic factors should also be considered. Sathyanarayanan et al. [19] found a statistically significant association between AGA and mean TC, mean high-density lipoprotein (HDL), and mean low-density lipoprotein (LDL), except for mean triglycerides [19]. Dyslipidemia seems to be associated with AGA and is more common among severe types of alopecia as compared to mild and moderate grades [17,19]. Interestingly, dyslipidemia occurs mainly in older AGA patients [20]. We believe the risk of dyslipidemia should be confirmed in larger sample studies, including more age-advanced patients with severe grades of alopecia.

## 5. Conclusions

Despite the fact that no significant differences were found in our study, we observed a possible link between lipid dysregulation and decreased concentrations of vitamin D and the development of alopecia. Therefore, it is important to screen patients with alopecia for metabolism-related markers.

No relationship was found between nutritional status, assessed using the BMI and the WHtR indices, and alopecia. In light of the conflicting reports in the literature, further research is necessary. Also, we found no correlation between gluten intolerance and alopecia areata.

We observed that the patients with alopecia were younger than the controls. We suggest that this might be associated with a modern lifestyle and the so-called ‘Western diet’. We are of the opinion that suboptimal nutrition and poor eating habits in childhood might constitute a risk factor for early hair loss in some individuals.

Although our knowledge and understanding of the role of micronutrients in non-cicatricial alopecia continue to increase, definitive clinical recommendations, e.g., routine laboratory testing or therapeutic supplementation, are not feasible at this point. Further multicenter studies with a large sample size and prospective design are necessary to elucidate the matter further. 

## Figures and Tables

**Table 1:** Types and etiologic factors of non-cicatricial alopecia.

Type of Non-Cicatricial Alopecia	Etiologic Factors
**Alopecia areata**	Specific autoimmune disease of the hair follicle Genetic background
**Tinea capitis (non-inflammatory type)**	Dermatophyte fungal infection
**Trichotillomania**	Hair-pulling
**Telogen effluvium**	Various factors such as drugs, physiological and emotional stress, and medical conditions leading to an abnormality in the normal hair cycle
**Anagen effluvium**	Chemotherapy-induced alopecia
**Androgenic alopecia**	Genetic causes

**Table 2:** Characteristics of the study group.

Types of Non-Cicatricial Alopecia in the Study Group												
Participants	Classification of Patients with AGA											
	Hamilton–Norwood						Ludwig					
	I n	II n	III n	IV n	V n	VI n	I n	II n	II n	TE n	AA n	Total n
all patients	1	3	3	4	4	1	3	3	1	10	4	37
male	1	3	3	4	4	1	0	0	0	0	1	17
female	0	0	0	0	0	0	3	3	1	10	3	20

Abbreviations: AGA—androgenetic alopecia; TE—telogen effluvium, AA—alopecia areata. n—number of patients.

**Table 3:** Distribution of anthropometric measurements in the study population.

Parameters Mean ± SD (Min–Max)	Study Population		*p*-Value
	Study Group	Controls	
Age (year)	40.46 ± 10.90 (21–62)	50.69 ± 8.41 (32–63)	0.0035 *
Waist circumference (cm)	85.81 ± 12.034 (68–115)	86.23 ± 10.29 (72–110)	0.9112
Weight (kg)	73.08 ± 16.59 (50–115)	72.00 ± 13.87 (51–94)	0.9382
BMI (kg/m^2^)	24.46 ± 4.75 (17.5–37.12)	26.01 ± 4.11 (18.96–33.74)	0.1773
WHtR	0.50 ± 0.06 (0.40–0.69)	0.52 ± 0.05 (0.45–0.63)	0.1635
Height (cm)	172.41 ± 7.02 (158–194)	166.23 ± 11.09 (153–187)	0.0791

Abbreviations: SD—standard deviation; WHtR—waist-to-height ratio, * significant difference.

**Table 4:** Distribution of the laboratory parameters in the study population.

Parameters (Mean ± SD)	Study Population		*p*-Value	Reference Range
	Study Group	Controls		
Hb (g/dL)	14.31 ± 1.15	14.03 ± 1.15	0.4633	13.7–17.5 male 11.2–11.7 female
WBC (K/uL)	6.39 ± 1.60	6.22 ± 2.17	0.7642	3.98–10.04
MCV (fL)	90.41 ± 4.43	90.48 ± 3.41	0.9629	79.04–94.08
TC (mg/dL)	200.03 ± 46.69	213.69 ± 45.78	0.3663	115–190
Vit. B_12_ (pg/mL)	405.62 ± 195.72	429.31 ± 128.62	0.1413	187–883
Ferritin (ng/mL)	96.06 ± 96.92	107.33 ± 151.04	0.6344	4.63–204
AST (U/L)	21.84 ± 9.92	21.69 ± 5.78	0.5644	5–34
ALT (U/L)	24.92 ± 22.23	26.54 ± 15.16	0.3816	0–55
Urea (mg/dL)	28.31 ± 6.10	28.87 ± 6.92	0.5652	21–43
Creatine (mg/dL)	0.82 ± 0.15	0.77 ± 0.14	0.3355	0.60–1.30
Triglycerides (mg/dL)	119.70 ± 74.44	110.85 ± 34.35	0.5802	0–150
Vitamin D25 (OH) (ng/mL)	27.42 ± 14.40	36.88 ± 23.43	0.0885	30–50
CRP (mg/L)	1.97 ± 2.42	1.83 ± 1.45	0.1796	0–5
Glucose (mg/dL)	89.30 ± 10.66	87.08 ± 13.94	0.5544	70–99
TSH (μlU/mL)	1.37 ± 0.68	1.03 ± 0.69	0.1269	0.35–4.94
DHEAS (ug/dL)	263.66 ± 134.95	193.42 ± 78.44	0.1326	139.7–484.4 male 56.2–282.9 female

## Data Availability

The original contributions presented in the study are included in the article/supplementary material, further inquiries can be directed to the corresponding author.
